# Mechanical Characterization of Core-Shell Rubber/Epoxy Polymers for Automotive Structural Adhesives as a Function of Operating Temperature

**DOI:** 10.3390/polym13050734

**Published:** 2021-02-27

**Authors:** Dooyoung Baek, Kyeng-Bo Sim, Hyun-Joong Kim

**Affiliations:** 1 Laboratory of Adhesion and Bio-Composites, Department of Agriculture, Forestry and Bioresources, College of Agriculture and Life Sciences, Seoul National University, Seoul 08826, Korea; baek.s.dy@snu.ac.kr (D.B.); skb181@snu.ac.kr (K.-B.S.); 2 Research Institute of Agriculture and Life Sciences, College of Agriculture and Life Sciences, Seoul National University, Seoul 08826, Korea

**Keywords:** toughened epoxy, core-shell rubber, adhesive, automotive, operating temperature

## Abstract

Automotive structural adhesives must show a steady toughness performance in the temperature range of −40 °C to 80 °C, considering their actual usage environments. Core-shell rubber (CSR) nanoparticles are known to enhance the toughness of epoxy systems. In this study, a CSR, pre-dispersed, diglycidyl epoxy of bisphenol A (DGEBA) mixture at 35 wt % (KDAD-7101, Kukdo Chemical, Seoul, Korea) was used as a toughener for an automotive structural epoxy adhesive system. A simple, single-component, epoxy system of DGEBA/dicyandiamide with a latent accelerator was adopted, where the CSR content of the system was controlled from 0 to 50 phr by the CSR mixture. To determine the curing conditions, we studied the curing behavior of the system by differential scanning calorimetry (DSC). Modulus variations of the cured bulk epoxies were studied using a dynamic mechanical analyzer (DMA) in the dual cantilever mode. The flexural modulus of the cured epoxies at various temperatures (−40, −10, 20, 50, and 80 °C) showed the same tendency as the DMA results, and as the flexural strength, except at 0 phr. On the other hand, the strain at break exhibited the opposite tendency to the flexural modulus. To study the adhesion behavior, we performed single-lap joint (SLJ) and impact wedge-peel (IWP) tests. As the CSR content increased, the strength of the SLJ and dynamic resistance to the cleavage of the IWP improved. In particular, the SLJ showed excellent strength at low temperatures (32.74 MPa at 50 phr @ −40 °C (i.e., an 190% improvement compared to 17.2 MPa at 0 phr @ −40 °C)), and the IWP showed excellent energy absorption at high temperatures (21.73 J at 50 phr @ 80 °C (i.e., a 976% improvement compared to 2.07 J at 0 phr @ 80 °C)). The results were discussed in relation to the changes in the properties of the bulk epoxy depending on the temperature and CSR content. The morphology of the fracture surface was also provided, which offered useful information for composition studies using the CSR/epoxy system.

## 1. Introduction

To respond to the annually strengthening carbon dioxide emission regulations [1,2,3] and improve the mileage range of electric vehicles that will replace internal combustion engine cars [4,5], the global automotive industry continues to trend toward weight reduction [6,7]. Diversified strategies, such as structural changes, material changes, and manufacturing process changes, are being considered for weight reduction [8,9,10,11]. Among these strategies, weight reduction by the reduction or exclusion of the classical mechanical fastening process has attracted attention, which involves the use of automotive structural adhesives [12,13,14,15,16].

In addition to achieving the purpose of weight reduction, structural adhesives have the following additional advantages [17]: uniform stress distribution on the fastening area by providing continuous bonding, improvement of fatigue resistance by minimizing the stress concentration, improvement of noise and vibration damping properties due to the relatively high energy absorption rate on the adhesive joints, securing the mechanical strength of the joint and protecting it from moisture and debris, adhesion between dissimilar materials, and prevention of the galvanic corrosion induced by intimate contact. Due to these various advantages, the number of structural adhesives consumed in manufacturing increases every year [18], and the significance of adhesive material contributions to automotive performance is emerging. Simultaneously, automobile and adhesive manufacturers have carefully considered and studied the reliability of adhesives in automobiles, where human safety must be guaranteed. The essential basic requirements for automotive structural adhesives are as follows: an elongation performance, in addition to the adhesive strength (i.e., the toughness) [19,20,21]; an impact resistance [22,23,24]; and a stable performance in the specified operating temperature range [25,26,27]. The toughness is required because a vehicle in the driving state experiences various external forces. The impact resistance is required to minimize the occupant’s impact by sufficiently absorbing or dispersing the impact on the vehicle body when a contact accident occurs. Finally, a stable performance in the operating temperature range is required to prevent catastrophic performance degradation at a specific temperature.

There are structural adhesives that are based on epoxy, urethane, and acrylic materials [19,26,27,28,29]. Among them, epoxy-based structural adhesives are commonly adopted because of their excellent mechanical strength, chemical resistance, and environmental resistance. However, since epoxy materials are generally brittle, the mechanical durability of an unmodified, epoxy adhesive against external forces, structural deformations, and impacts is weak. To overcome this brittleness and improve their automotive applicability, it is common to improve their toughness using the following methods: formulating epoxy adhesives with urethane or rubber-based additives, adding thermoplastic or inorganic filler particles, and cross-linking with synthesized epoxy that has a urethane or rubber molecular structure. Core-shell rubber (CSR) nanoparticles are among the most widely used additives for improving the toughness of epoxy polymers since their shells and cores are made of thermoplastic and rubber-based materials, respectively [23,24,30,31,32,33,34,35].

Quan and Ivankovic [35] investigated CSR nanoparticle/epoxy composite polymers using a diglycidyl epoxy of bisphenol A (DGEBA)/dicyandiamide (DICY) epoxy system. They used two sizes of CSR nanoparticles (i.e., 203 and 74.1 nm) and found an optimum CSR content of 30 vol %, which improved the fracture energy from 343 J/m^2^ of neat epoxy up to 2671 J/m^2^. Quan et al. [36] also used CSR in epoxy adhesives and found that the CSR addition changed the failure mode of the single lap joint test (SLJ test) from brittle and interfacial to ductile and cohesive. Back et al. [23] and Chae et al. [24] also used CSR in automotive structural adhesives and characterized its impact performance using the impact wedge-peel test (IWP test, ISO 11343 [37]), since the IWP test is suitable and preferrable for automotive structural adhesives that require high impact resistance under high-speed impacts with high energies [22]. However, conventional studies on CSR nanoparticle/epoxy composite polymers have mainly considered the mechanical properties at room temperature. A number of studies have characterized the properties of the CSR/epoxy at cryogenic temperatures (at 77 K under LN_2_ ambient conditions) [38,39] and low temperatures (from −109 to 20 °C) [40,41,42], but these studies are rare. Nevertheless, this temperature range is only a partial intersection of the operating temperature range required for the automotive industry, which presents insufficient information for structural adhesive manufacturing. To apply CSR to the adhesives, one must provide and discuss property characterization in the operating temperature range. In general, the operating temperature range of adhesive joints used in the automotive industry is −40 to 80 °C [25,26,27]; thus, adhesion performance tests, such as SLJ and IWP tests, must be conducted and discussed in this temperature range to provide useful information for performance evaluation.

Conventional studies dealing with the influence of temperature on the adhesion performance of structural adhesives have tended to use commercial structural adhesives. Banea et al. [26] used two commercial, automotive epoxy and polyurethane structural adhesives and characterized their tensile and adhesion properties at −40 to 80 °C. They found that the tensile and shear strengths varied with temperature. Na et al. [27] used a commercial, automotive, epoxy adhesive and characterized its tensile and adhesion properties at −40 to 80 °C. They found that, as the temperature increased, the Young’s modulus and tensile strength decreased. Additionally, the tensile strain increased, and the mechanical properties changed significantly as the glass transition temperature was approached or exceeded. Da Silva et al. [28] considered the application of adhesive joints in supersonic aircraft and characterized the tensile and shear properties of three commercial, structural adhesives at −55 to 200 °C. They suggested that the correlations between the tensile and shear were reasonable in terms of stiffness and strength but were poor in terms of ductility. These studies showed that the characterization of material properties in a specific temperature range was significant, considering the industrial applications.

The aims of this study were to investigate the influence of the operating temperature on the bulk flexibility, adhesion strength, and high-speed impact resistance of CSR nanoparticles/epoxy adhesives with different CSR contents. Basic thermal characterizations of the curing and modulus were performed using differential scanning calorimetry (DSC) and dynamic mechanical analysis (DMA). The DGEBA/DICY epoxy system was adopted as the main component of the adhesives; the CSR contents were set to 0, 10, 20, 30, 40, and 50 phr (parts per hundred resin); and the ambient temperatures for characterization were set to −40, −10, 20, 50, and 80 °C. Each of the three above-mentioned properties was characterized by a three-point flexural test, SLJ test, and IWP test under each set temperature. The morphology of the fracture surface was studied by field emission scanning electron microscopy (FE-SEM) and optical microscopy (OM).

## 2. Materials and Methods

### 2.1. Materials

DGEBA (YD-128, Kukdo Chemical Co., Ltd., Seoul, Korea) and DICY (DYHARD 100S, AlzChem Group AG, Trostberg, Germany) were used as the main components of the structural adhesives. Considering their application as a one-part, we used epoxy adhesive, an aromatic-substituted urea accelerator (OMICURE U-405, Huntsman Corporation, The Woodlands, TX, USA). The CSR, pre-dispersed, DGEBA resin (35 wt %) (KDAD-7101, Kukdo Chemical Co., Ltd.) was used to control the CSR content of the epoxy adhesives. The shell and core of the CSR nanoparticles (200 to 450 nm diameter) were composed of poly (methyl methacrylate) (PMMA) and butadiene rubber, respectively [23]. Ground calcium carbonate (GCC (CaCO_3_), Omyacarb 10, Omya AG, Oftringen, Switzerland) was used as the filler for the basic performance as an adhesive [23,24,43,44]. Table 1 shows the details of each component.

A cold-rolled, high-strength, steel sheet with a thickness of 1.6 mm, SPFC340 (CHSP35R, POSCO, Pohang, Korea), was used as an adherend in the SLJ and IWP tests. SPFC340, also recognized as SPFC340 (KS and JIS), A1008-33 (ASTM), and HC 220P (EN), is classified as an automobile structural steel with improved formability for the metalworking of drawing and stamping. SPFC340 was cut to dimensions of 25 × 100 mm^2^ for the SLJ test and 20 × 90 mm^2^ for the IWP test. Before applying structural adhesives, we dried all adherends at 20 °C and 55% relative humidity (RH) for 1 h after cleaning with isopropyl alcohol and clean cloths.

### 2.2. Composition of CSR/Epoxy Polymers for Structural Adhesives

The total amount of DGEBA, consisting of YD-128 and KDAD-7101 (65 wt %), was set to the hundred resin, and the CSR mixture was added to control the CSR content at 0, 10, 20, 30, 40, and 50 phr, as shown in Table 2. The DICY hardener was added with a 1:1 equivalent weight ratio of epoxy and amine, and the substituted urea accelerator and GCC were added at contents of 1 phr and 3 phr, respectively. Since the CSR content of KDAD-7101 is 35 wt %, 53.85 phr (31.85 wt %) is the upper limit of the CSR content, and thus 50 phr was set to be the maximum content. In addition, the CSR contents relative to the total weight (wt %) are provided in Table 2.

Each composition was mixed using a paste mixer (ARV-310, Thinky, Tokyo, Japan) according to the following procedure: (i) the CSR mixture was preheated to 50 °C for ease of work; (ii) the DGEBA and CSR mixture was mixed for 3 min at 2000 rpm (under a 1.0 kPa vacuum); (iii) the DICY, accelerator, and GCC (powder type) were added to the mixture; (iv) the mixture was mixed for 2 min at 2000 rpm (under atmospheric pressure) to prevent scattering of the powdered compositions; and then (v) the mixture was further mixed for 5 min at 2000 rpm (under a 1.0 kPa vacuum) for dispersion and defoaming.

### 2.3. Curing Condition Determination by Differential Scanning Calorimetry

To determine the curing temperature and time required for the CSR/epoxy adhesives, we evaluated the thermal behavior of each composition by DSC (DSC Q200, TA Instruments, New Castle, DE, USA). An aluminum pan and lid were used, and the mass of each sample in the pan was controlled to be 12 ± 1 mg. The heat flows in the exothermic curing reaction were measured under a temperature sweep from 50 to 250 °C at a constant heating rate of 5 °C/min for the curing temperature determination. On the basis of the temperature sweep results, we determined a common value close to the temperature at which the heat flows reached the maximum value as the curing temperature. Moreover, the isothermal heat flow behaviors at the curing temperature for 60 min were measured for the curing time determination.

### 2.4. Dynamic Mechanical Analyzer

To characterize the modulus and tan *δ* behaviors of the bulk materials of the CSR/epoxy adhesives under the operating temperature range (−40 to 80 °C), we performed the dual cantilever mode by DMA (DMA Q800, TA Instruments) with an oscillation strain of 0.1%, frequency of 1 Hz, and temperature ramp from −50 to 200 °C at a constant heating rate of 5 °C/min.

The DMA specimens were fabricated by casting the CSR/epoxy adhesives on an aluminum mold (80 × 10 × 4 mm^3^). The CSR/epoxy adhesives were placed in a syringe in a state preheated to 50 °C to improve their flowability and prevent bubble formation during casting, and were carefully cast in the mold preheated to 120 °C. The bulk adhesives in the mold were cured at 150 °C for 30 min. The cured bulk adhesives were released from the mold after they cooled down to 20 °C.

### 2.5. Three-Point Flexural Test

The adherend cannot be assumed to be a rigid body in the structural adhesion tests; therefore, a bending moment was applied in the adhesive joints, during which the joints were deformed or fractured. This shows that the stress distribution on the adhesive joints is a complex system that does not consist of pure tensile or compressive stress. These are well described in adhesive joint studies using a finite element method [45]. Therefore, in order to evaluate the strength and elongation of the bulk materials of the CSR/epoxy adhesives, we adopted a three-point flexural test in which the tensile and compressive stresses worked together. The specimens for the test were fabricated at the same size and condition as the DMA specimens. The test was conducted using a universal testing machine (UTM) (Z010 with a 15 kN load cell and a custom chamber for ambient temperature control (−170 to 300 °C), Zwick/Roell Group, Ulm, Germany) following ISO 178. Therefore, the span was 64 mm with supporting and loading pins that had radii of 5 mm; the flexural modulus (*ε* ≤ 0.25%) and the strain–stress curves (*ε* ≥ 0.25%) were measured at crosshead speeds of 2 mm/min and 10 mm/min, respectively.

### 2.6. Single-Lap Joint Test

**Figure 1:**
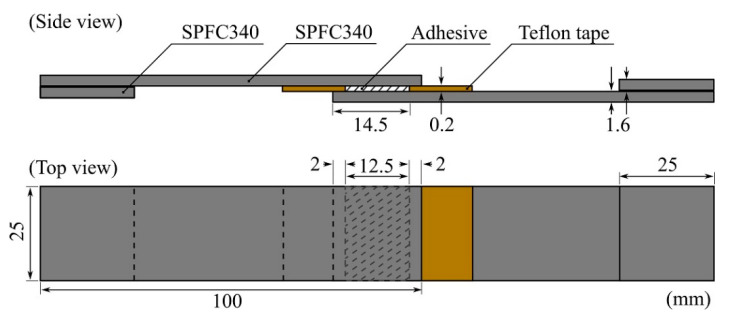
Schematic illustration of the specimen for the single-lap joint (SLJ) test. The Teflon tapes were attached at 14.5 mm from the end tips, and the two adherends were overlapped at an adhesive length of 12.5 mm after the adhesive application. Grip tips with lengths of 25 mm were attached to each side for alignment.

An SPFC340 sheet with a thickness of 1.6 mm was cut to dimensions of 25 × 100 mm^2^ for the SLJ test. Before application of the adhesives, the adherends were cleaned with isopropyl alcohol with clean cloths and dried at 20 °C and 55% RH for 1 h. To ensure the adhesive thickness, we attached a Teflon glass cloth tape (width of 18 mm, thickness of 0.2 mm, AGF-100FR, Chukoh Chemical Industries, Ltd., Tokyo, Japan) at 14.5 mm from the end tips of all adherends. After applying the adhesive to the bonding line using a wooden stick, we formed a 12.5 × 25 mm^2^ bonding area with a thickness of 0.2 mm by overlapping the end tips to the attached Teflon tape 2 mm apart from each other, as shown in Figure 1. The constructed specimens were cured at 150 °C for 30 min using a fan-assisted oven. The SLJ test was conducted using the UTM with a crosshead speed of 5 mm/min.

### 2.7. Impact Wedge-Peel Test

**Figure 2:**
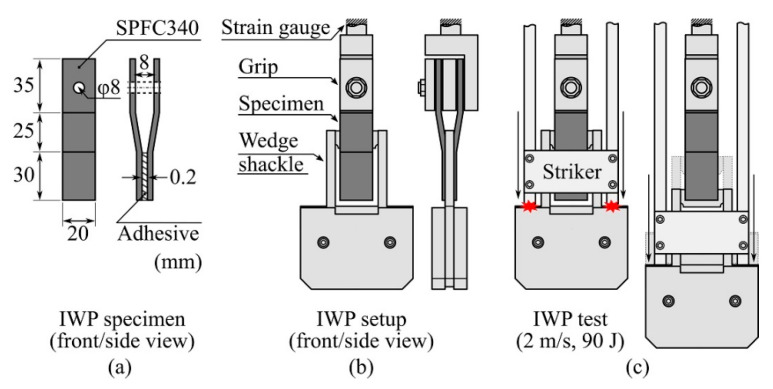
Schematic illustration of the (**a**) impact wedge-peel (IWP) specimen and dimension of each IWP adherend; (**b**) IWP setup in the drop tower chamber; (**c**) initial impact of the striker to the wedge shackle shoulder (**left**); and impact cleavage process (**right**) during the IWP test.

An SPFC340 sheet with a thickness of 1.6 mm was cut to 20 × 90 mm^2^ for the IWP test; stamping, bending, and punching were performed to obtain IWP adherends, as shown in Figure 2a. Before application of the adhesives, the adherends were cleaned with isopropyl alcohol with clean cloths and dried at 20 °C and 55% RH for 1 h. To ensure the adhesive thickness, we used a small amount of glass beads, which had diameters of 0.2 mm (UNITECH, Ansan, Korea), after applying the adhesive to the bonding line using a wooden stick. A 30 × 20 mm^2^ bonding area with a thickness of 0.2 mm was formed, as shown in Figure 2a. The constructed IWP specimens were cured at 150 °C for 30 min using a fan-assisted oven.

The IWP test was conducted using a drop tower (CEAST 9350 with an environmental chamber system (−70 °C to 150 °C), Instron, Norwood, MA, USA). As shown in Figure 2b, the wedge shape (with an edge radius of 1 mm) installed between the gaps in the IWP specimen complied with ISO 11343. The drop tower recorded and output the change over time in the force applied to the specimen and the displacement of the striker (Figure 2c) during the IWP test. The striker’s total weight was set to 45 kg, and the striker’s drop height was set to reach a speed of 2 m/s at the initial impact with the wedge shackle shoulder (Figure 2c, left), such that the energy received by the IWP specimen at impact was 90 J.

### 2.8. Morphology of Fractured Cross-Section

The morphology of the fractured cross-section was studied using FE-SEM (SUPRA 55VP, Carl Zeiss, Oberkochen, Germany) and OM (SV-55, SOMETECH INC., Seoul, Korea). All SEM images were captured using setting values as follows: magnification (30,000×); electron high tension (EHT = 2.0 kV); detector (InLens).

## 3. Results and Discussion

### 3.1. Curing Behavior and Curing Condition Determination

**Figure 3:**
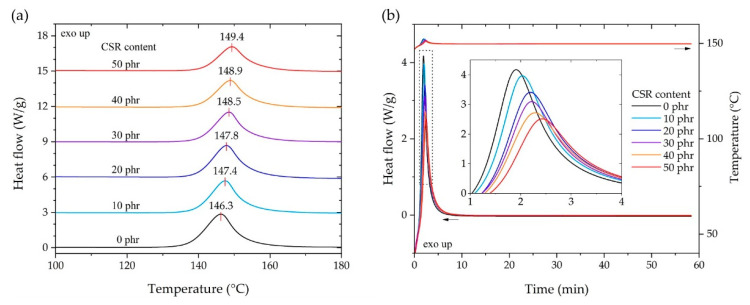
Exothermic curing reaction of the differential scanning calorimetry (DSC) results according to the content of CSR nanoparticles. (**a**) Temperature sweep from 50 to 250 °C at a constant heating rate of 5 °C/min. (**b**) Isothermal heat flow behaviors at 150 °C.

Figure 3a shows the heat flow of each composition in the exothermic curing reaction under a temperature sweep. As the CSR content increased, the temperature of each exothermic curing peak improved in the range of 146.3 to 149.4 °C. The peak temperature was observed when the crosslinking reaction between DGEBA and DICY was violent; considering that the temperature rate was constant, it was suggested that the crosslinking reaction was delayed depending on the content of CSR nanoparticles. However, even when the CSR content reached 30% of the total weight composition ratio (Table 2), the peak temperature change of 3 °C showed a relatively small delay compared to the toughened epoxy system using rubber-modified epoxy (RME) or urethane-modified epoxy (UME), as reported by Back et al. [44]. From the results in Figure 3a, the curing temperature was determined to be 150 °C and, as shown in Figure 3b, the exothermic behavior under isothermal conditions was observed to determine the curing time. For the isothermal, exothermic behavior, it was clearly observed that the CSR content delayed the reaction rate in the range of 1.8–2.5 min on the basis of the peak of heat flow. All heat flows converged to the flat area at 15 min and no exothermic reaction was observed. Considering the above results and the curing environment using the aluminum molder and SPFC340 adherends, the curing temperature and time were determined to be 150 °C and 30 min, respectively.

### 3.2. Bulk Properties

**Figure 4:**
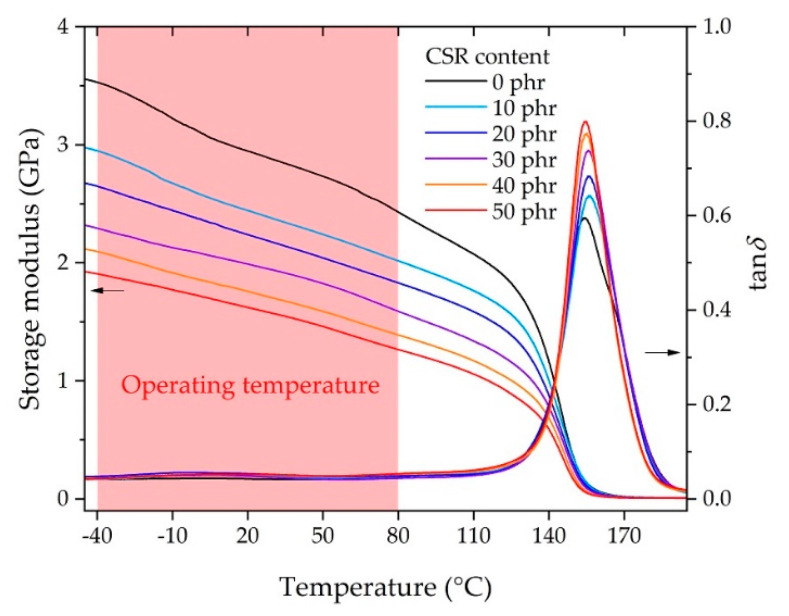
Storage modulus and tan *δ* measured by dynamic mechanical analyzer (DMA).

Figure 4 shows the DMA results of the bulk adhesives according to the CSR content in the dual cantilever mode. It shows the change in the storage modulus and tan *δ* according to the temperature sweep. The storage modulus decreased as the CSR content increased in the measurement temperature range. In the operating temperature range, it was observed that the storage modulus decreased linearly with increasing temperature, and it was verified that the coefficient of determination (*R*^2^) was 0.99 for all curves when performing linear fitting in the temperature range. The storage modulus started to decrease sharply from 120 to 155 °C, near the glass transition temperature *T*_g_ (determined by the peak of the tan *δ* curve), which suggested the ease of design of the adhesive according to the CSR content at the operating temperature. The *T*_g_ values were distributed from 154.4 to 156.4 °C, and an improvement in the damping ratio was observed because tan *δ* at *T*_g_ increased with the increase in the CSR content.

**Figure 5:**
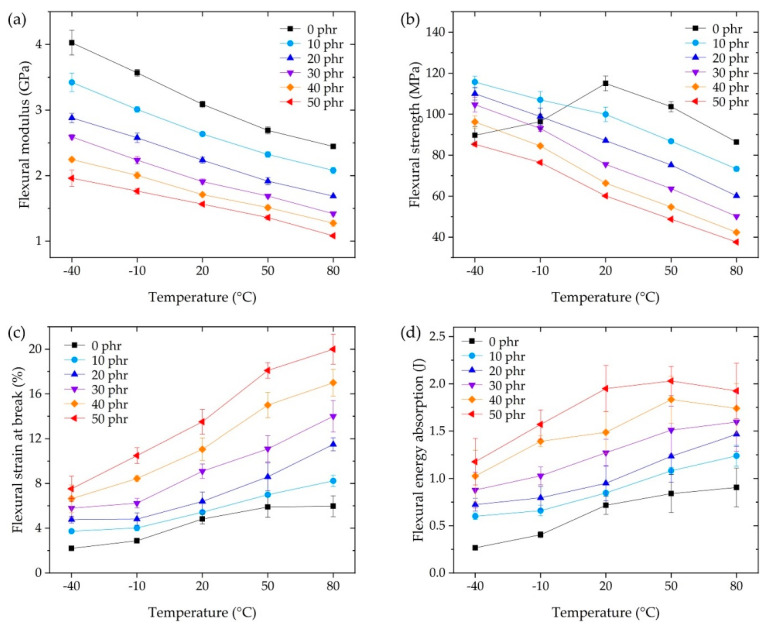
Three-point flexural tests at the operating temperature, showing the (**a**) flexural modulus, (**b**) flexural strength, (**c**) flexural strain at break, and (**d**) flexural energy absorption.

Figure 5 shows the flexural modulus, strength, strain at break, and flexural energy absorption at the operating temperature for each CSR content. The flexural modulus was calculated following ISO 178. The flexural strength and strain at break were selected from the maximum stress and strain at rupture in each measured stress–strain curve. The flexural energy absorption was obtained by integrating each force–displacement curve from the start of the test to rupture.

The flexural modulus in Figure 5a showed the same tendency as the storage modulus (Figure 4), although the measurement conditions were different, i.e., (DMA: dynamic) the dual cantilever, 1 Hz oscillation with 0.1% strain, and 5 °C/min temperature rate, versus (flexural: static) three-point flexural, 2 mm/min deformation rate for 0.25% strain, and a 30 min stabilization duration for each sample at the temperature. The modulus of the CSR core was negligible (less than 1%) compared to the shell and the DGEBA/DICY cross-linked matrix because the modulus of PMMA (i.e., the CSR shell) was reported to be in a similar range of 0 phr epoxy at 2–4 GPa [46,47], whereas the modulus of butadiene rubber (i.e., the CSR core) was in the range of 1–10 MPa [35]. Therefore, the volumetric fraction of the DGEBA/DICY matrix per unit volume decreased and the apparent modulus decreased as the CSR content increased due to the contribution of the rubbery phase of the CSR core. Depending on the CSR content, from 0 phr to 50 phr, the modulus decreased up to 45% (Figure 5a); similarly, the strength decreased up to 55% (Figure 5b), while the flexural strain at break increased up to 270% (Figure 5c). This showed that the improvement in strain at break was greater than the loss of modulus or strength due to the addition of CSR; these simple tendencies in the results suggested the ease of the adhesive design at the operating temperature. Notably, the flexural strength of 0 phr at −40 and −10 °C that did not follow the tendency of the other results was the point where the rupture occurred earlier than the strength vertex, representing the brittleness of the base matrix (0 phr) in the low temperature range. This brittleness seemed to be overcome by a CSR content of 10 phr, but it should be noted that the improvement through the use of the filler was not an improvement of the matrix itself. Since the outline of the energy absorption in Figure 5d was similar to that of Figure 5c, it can be understood that the change in the performance of the CSR content contributed to significantly increasing the strain at break, as discussed above.

**Figure 6:**
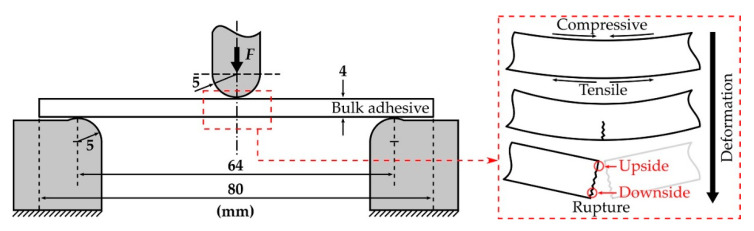
Schematic illustration of the three-point flexural test (**left**) and bulk adhesive specimen deformation and rupture (**right**).

In the three-point flexural tests, tensile and compressive stresses were applied to the downside and upside of the bulk adhesive, respectively, as shown in Figure 6. When the specimen could no longer withstand the deformation caused by the loading pin, rupture instantaneously occurred. Since the specimen was unnotched, it was difficult to assert that cracks occurred at some point on the downside or upside. However, it has been reported that the fracture stress and strain of CSR/epoxy composites are weaker under tensile conditions than under compression [35]. Therefore, we assumed that the rupture first occurred on the downside of the specimen under tensile stress, and the strain energy stored in the bulk adhesive was released by the crack propagation that was instantaneously performed on the upside under compressive stress.

**Figure 7:**
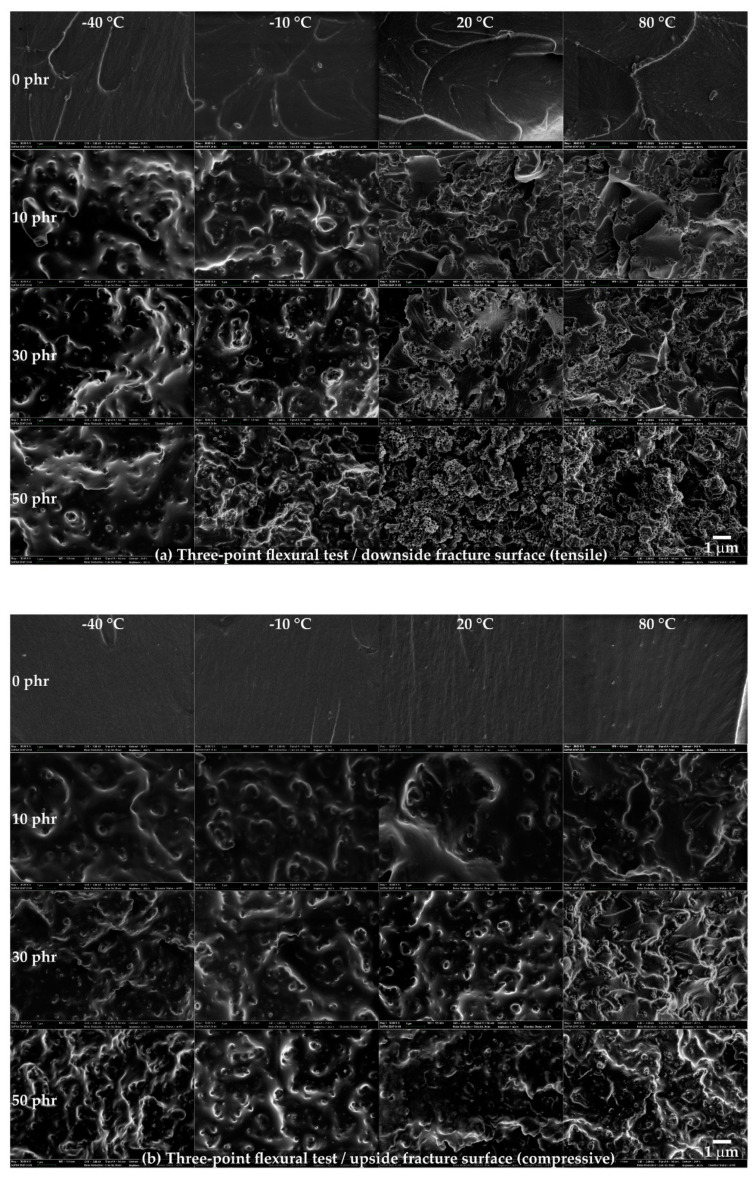
Field emission scanning electron microscopy (FE-SEM) images of the fracture surface in the three-point flexural tests.

**Figure 8:**
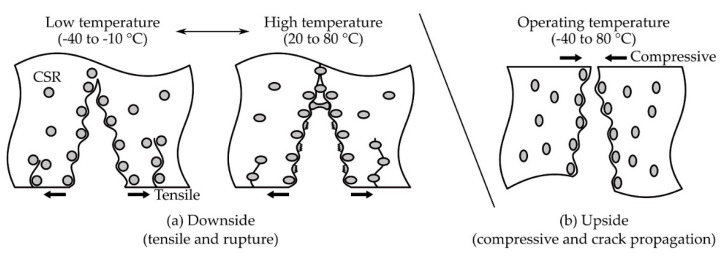
Schematic illustration of the rupture and crack propagation mechanism on the bulk adhesive with CSR content in the three-point flexural test. (**a**) Downside and (**b**) upside surfaces.

The fracture surfaces on the downside and upside were observed using FE-SEM (Figure 7). On the downside fracture surface (Figure 7a), at 0 phr, a rougher surface was observed at higher temperatures. From 10 phr, a random, spin-shaped, fracture surface was observed at −40 and −10 °C, and spherical, pore-shaped surfaces began to be observed after 20 °C. In addition, it was observed that the density of the spin-shaped (−40 to −10 °C) and spherical, pore-shaped (20 to 80 °C) surfaces increased as the CSR content increased. On the upside fracture surface (Figure 7b), on the other hand, a smooth surface was observed at 0 phr at the operating temperature. As the CSR content increased, a spin-shaped, rough, fracture surface was observed, but a spherical, pore-shaped, fracture surface observed at the downside from 20 to 80 °C (Figure 7a) was not observed at the upside. The spin-shaped and spherical, pore-shaped surfaces, which were not observed at 0 phr but were observed from 10 phr, suggested that this phenomenon was derived from the CSR nanoparticles. In addition, the fact that different fracture shapes were observed depending on the temperature range at the downside suggested that the brittleness of 0 phr at −40 and −10 °C, observed in Figure 5b, may have been the dominant factor in determining the fracture shape. Figure 8 illustrates these fracture shapes by considering the micromechanical mechanism proposed by Pearson and Yee [48]. At low temperatures on the downside surface (Figure 8a (left)), the brittleness of the matrix may have been higher than that of the CSR shell, and the deformation transfer between the matrices was relatively easy compared to that between the matrix and CSR; therefore, it was assumed that the selective rupture caused at the matrix consumed relatively little energy. At high temperatures on the downside surface (Figure 8a (right)), the ductility of the matrix increased and, as the deformation transfer between all components became easier, CSR particle yielding occurred as a result. It was assumed that the rupture occurred around the yielded point. In the case of the upside surface, the occurrence of downside rupture triggered the instantaneous release of the strain energy accumulated in the bulk, and the crack propagated to the upside surface where compression was acting. Since cracks in a different direction from the deformation instantaneously propagated, it was assumed that fracture occurred at the matrix and CSR/matrix interface where crack propagation was relatively easy. It should be noted that these fracture shapes were observed only under certain stress, strain, and velocity conditions of the three-point flexural test and may be difficult to apply universally to any system with different failure conditions.

### 3.3. Adhesion Performances

#### 3.3.1. Single-Lap Joint Test

**Figure 9:**
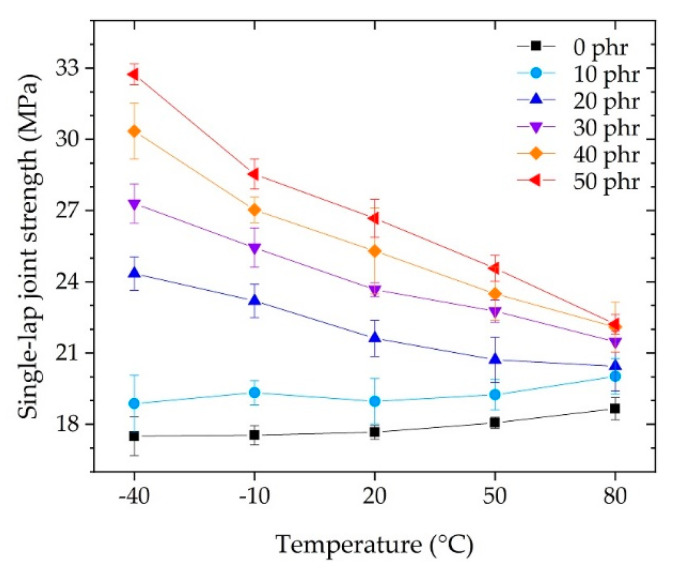
Strength of single-lap joint (SLJ) tests as a function of operating temperature.

The SLJ test is a test under relatively static conditions and is used as a measure of adhesion performance to determine how much load the unit bonding area can withstand. In addition, it is possible to evaluate the unit elements composed of the adhesive and adherend with a specified thickness and material used in the automotive industry. Figure 9 shows the strength measured in the SLJ test. At each temperature, the SLJ strength increased as the CSR content increased. At each CSR content, the SLJ strength decreased as the temperature increased. For each temperature, referring to the flexural modulus of Figure 5a or the flexural strength of Figure 5b, it was expected that the SLJ strength would decrease as the CSR content increased. However, contrary to expectations, the SLJ strength increased as the bulk elongation increased depending on the flexural strain at break, as shown in Figure 5c. For each CSR content, the SLJ strength decreased as the temperature increased, depending on the flexural modulus of Figure 5a, except for the cases of 0 phr and 10 phr, which showed slight fluctuations.

**Figure 10:**
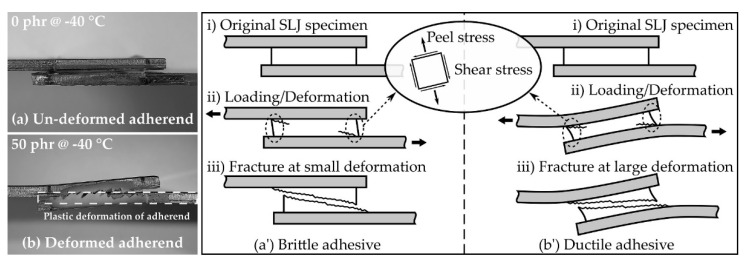
Side view of the fractured SLJ specimens overlapped manually for photographs for the (**a**) un-deformed adherend (0 phr @ −40 °C) and (**b**) deformed adherend (50 phr @ −40 °C). Schematic illustration of SLJ deformation on the (**a’**) brittle adhesive and (**b’**) ductile adhesive.

Since the SLJ specimen was asymmetrical with respect to the axis of force action, the tensile stress and bending moment acted on the SLJ specimen [49]; as a result, the shear and peel stresses acted on the adhesive, as shown in Figure 10. Moreover, since the applied adhesive had a finite length and had both end tips, the stresses acting on the adhesive were maximized at the tips [50,51,52]. These stresses caused deformation of the adhesive and, in the case of the SLJ test, it was possible to qualitatively examine the deformation resistance characteristics of the adhesive by observing the appearance of the specimen after the test. Figure 10a,b shows side views of the SLJ specimen after each test at 0 phr and 50 phr, respectively, at −40 °C. In the case of 0 phr, the specimen was fractured without deformation (Figure 10a); whereas, in the case of 50 phr, it fractured with bending plastic deformation (Figure 10b). This deformation was a result of the brittle (Figure 10a’) and ductile (Figure 10b’) properties of the adhesive for excessive and complex stress–strain conditions occurring locally on both end tips. The above discussion suggested that the bulk elongation (Figure 5c) was an important factor, in addition to the bulk modulus and strength of the adhesive, for the development of adhesive performance under static conditions such as the SLJ test.

**Figure 11:**
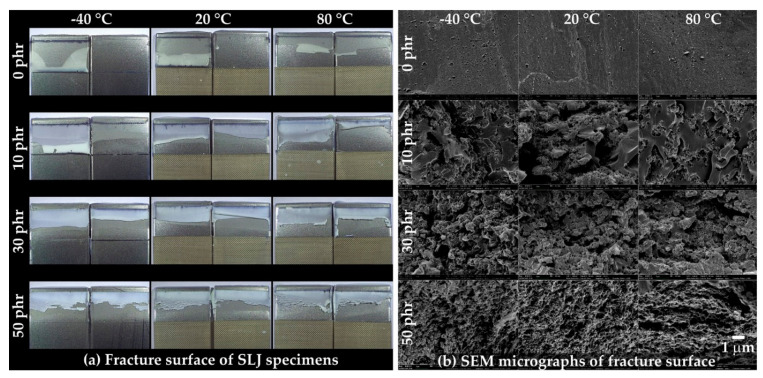
(**a**) Fracture surface of the SLJ specimens captured by optical microscopy (OM) and (**b**) FE-SEM micrographs of the fracture surface.

The fracture surfaces of the SLJ specimens were observed using OM (Figure 11a) and FE-SEM (Figure 11b). For the OM shown in Figure 11a, a rougher fracture surface was observed as the CSR content increased and the temperature increased. Except for at 0 phr (−40 to 20 °C), the residual adhesive on the top of each pair of fractured specimens suggested that crack propagation generally occurred at the point where the bending deformation of the specimen was excessive (Figure 10b’(ii)). In the FE-SEM image shown in Figure 11b, a relatively smooth fracture surface was observed at 0 phr, and the spherical, pore-shaped, fracture surface began to be observed at 10 phr. In addition, as the CSR content increased, the spherical, pore-shaped density and roughness were observed. Unlike Figure 7a, the observation of spherical pore-shaped surfaces at all temperature ranges may have been due to deformation fracture under complex stress consisting of shear and peel.

#### 3.3.2. Impact Wedge-Peel Test

**Figure 12:**
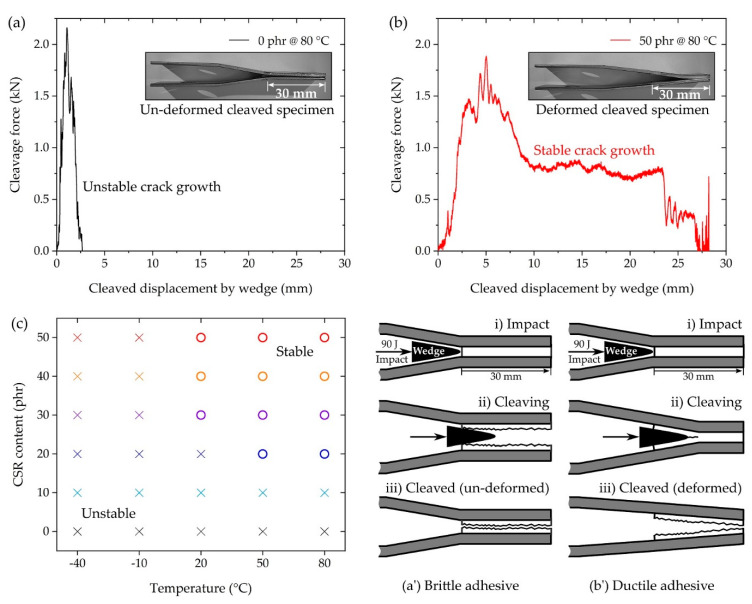
IWP test results depicting (**a**) unstable crack growth (0 phr @ 80 °C), (**b**) stable crack growth (50 phr @ 80 °C), and a (**c**) stable–unstable map of all compositions. Schematic illustration of the IWP specimen cleavage and deformation on a (**a’**) brittle adhesive and a (**b’**) ductile adhesive.

In the IWP test, a striker applied an impact energy of 90 J to the shoulder of the wedge shackle, and the wedge cleaved the IWP specimen (Figure 2c and Figure 12a’,b’). The results measured in the IWP test were classified into unstable crack growth (Figure 12a) and stable crack growth (Figure 12b), according to ISO 11343, from the shape of the cleavage force–displacement plot. In ISO 11343, the dynamic resistance to cleavage (N/mm, cleavage force per adhesive width of 20 mm) was calculated for the quantification of the IWP test. This value was calculated from the plateau region shown in Figure 12b, and therefore the result of not measuring the plateau region, as shown in Figure 12a, was classified as unstable. The results of the stable–unstable classification of the IWP test by temperature and CSR content are shown in Figure 12c, where only the tests above 20 °C and 20 phr were classified as stable (except the case of 20 phr @ 20 °C). Unlike the SLJ test, which applied a constant strain rate using the UTM, in the IWP test, an instantaneous force and deformation were applied to the adhesive by wedge impact. In the case of brittle adhesives, which do not exhibit resistance to such an instantaneous force and deformation, instantaneous fracture was caused by initial crack propagation, as shown in Figure 12a’. As a result, the IWP-adherend specimen did not undergo deformation (Figure 12a). On the other hand, in the case of the ductile adhesive, as shown in Figure 12b’, there was resistance of the adhesive to the instantaneous crack propagation initiated by the wedge and, as a result, the IWP-adherend specimen was accompanied by deformation (Figure 12b). Considering the poor elongation (Figure 5c) and SLJ strength (Figure 9) of 0 phr and 10 phr, the unstable result of the IWP test was reasonable. The unstable results at −40 and −10 °C suggest that the low-temperature, brittle characteristics of the base matrix (0 phr), discussed in Figure 5b, acted predominantly under the dynamic conditions of the IWP test, regardless of the CSR content.

**Figure 13:**
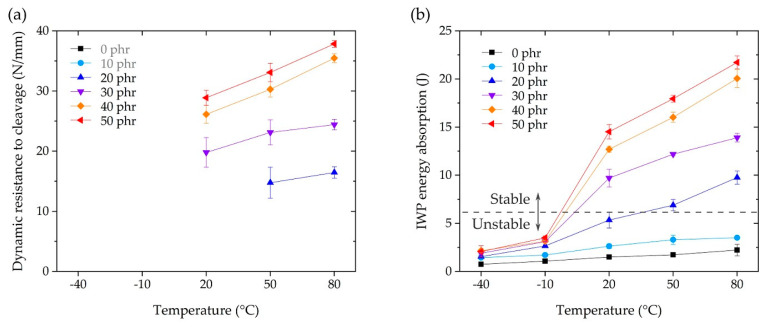
(**a**) Dynamic resistance to cleavage and (**b**) IWP energy absorption as a function of the operating temperature.

As mentioned above, dynamic resistance to cleavage can only be calculated in the IWP test when it is classified as stable crack growth (Figure 12b,c), where the calculation result for stable growth is shown in Figure 13a. The improvement in dynamic resistance to cleavage as the temperature increased was similar to the variation in bulk adhesive elongation (Figure 5c) and toughness (Figure 5d). The IWP energy absorption in Figure 13b is the result of calculating the area under the cleavage force–displacement curve, e.g., Figure 12a,b. In the case of 50 phr of energy absorption compared to 0 phr, the absorption at −40 and −10 °C improved by 277% and 326%, respectively. At 20 °C, the absorption increased sharply to 967%, and at 50 and 80 °C, the improvements were 1037% and 976%, respectively. In Figure 13, the boundary between the stable and unstable crack growth is indicated by a dotted line, which suggests that a stable IWP test could be obtained at an energy absorption of more than 6 J. These results suggested that the effective temperature of the CSR/epoxy system for high-speed impact resistance was in the range of 20 to 80 °C, and the CSR content necessary for stable performance was more than 30 phr.

**Figure 14:**
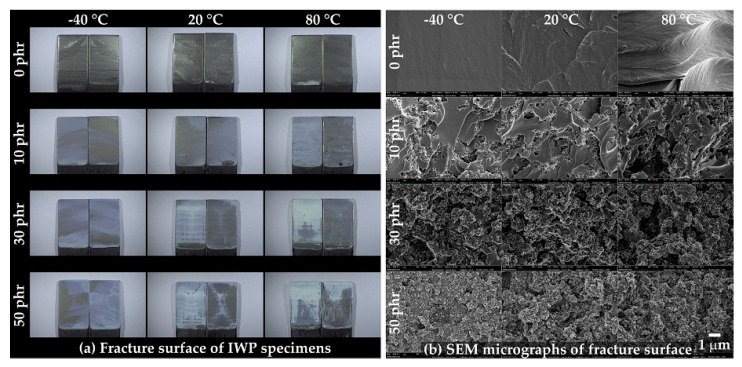
(**a**) Fracture surfaces of the IWP specimens captured by OM and (**b**) SEM micrographs of the fracture surfaces.

The fracture surfaces of the IWP specimens were observed using OM (Figure 14a) and FE-SEM (Figure 14b). As shown in Figure 14a, cohesive failure was observed in all compositions, and rougher fracture surfaces were observed at high temperatures and high CSR contents. The same roughness tendency was observed in the FE-SEM image, shown in Figure 14b, and, similar to Figure 11b, the spherical, pore-shaped, fracture surface began to be observed from 10 phr.

## 4. Conclusions

For the purpose of automotive, structural adhesive applications, we investigated the behavior of a CSR nanoparticle/epoxy system as a function of operating temperature. For CSR contents up to 50 phr, as a function of the operating temperature, the results of a three-point flexural test, SLJ test, and IWP test showed a monotonous tendency to increase or decrease, indicating the ease of use of CSR for the epoxy system. It was possible to improve the elongation of the CSR/epoxy system by adding CSR (Figure 5c), but since CSR is a nanoparticle-type filler, it was impossible to modify the inherent brittleness of the base matrix (0 phr) itself. In the SLJ test, under fixed temperature conditions, the expression of SLJ strength was related to the variation in elongation (Figure 5c) according to the CSR content. Under fixed CSR content conditions, the expression of SLJ strength was related to the variation in the modulus (Figure 5a) according to temperature. A maximum SLJ strength of 32.7 MPa (Figure 9) was obtained at 50 phr @ −40 °C (i.e., a 190% improvement compared to 17.2 MPa at 0 phr @ −40 °C), which decreased with increasing temperature; a strength of 22.2 MPa was obtained at 50 phr @ 80 °C (i.e., a 119% improvement compared to 18.66 MPa at 0 phr @ 80 °C). In the IWP test, stable crack growth results were obtained only above 20 °C and 20 phr (excepting the case of 20 phr @ 20 °C). For the stable results, higher dynamic resistance to both cleavage and energy absorption (Figure 13) was obtained with increasing CSR content and temperature. The results suggested that the effective CSR/epoxy system for applications is limited to 20 °C (30 phr) or higher, and that it is essential and significant to consider the operating temperature in composition studies. In future work, we will explore additives or networks that improve dynamic impact properties at −40 °C for epoxy-based systems and discuss a composition mechanism for exerting the properties at low temperatures. Moreover, the fracture toughness and fracture surface roughness will be considered through a single edge notch bend test to discuss fracture mechanics.

## Figures and Tables

**Table 1:** Components of structural adhesives.

Material		Code	Abbreviation	Equivalent Weight, g/eq
Resin	Diglycidyl ether of bisphenol A	YD-128	DGEBA	187
	CSR dispersed DGEBA at 35 wt %	KDAD-7101	CSR mixture	287.7
Hardener	Dicyandiamide	DYHARD 100S	DICY	21
	Aromatic substituted urea	OMICURE U-405	Accelerator	-
Filler	Ground calcium carbonate	Omyacarb 10	GCC	-

**Table 2:** Composition of the core-shell rubber (CSR)/epoxy polymer structural adhesives.

CSR Content ^1^, phr	DGEBA (YD-128), g	CSR Mixture, g			DICY (100S), g	Accelerator (U-405), g	GCC, g	CSR, wt %
		**KDAD-7101**	**DGEBA**	**CSR**				
0	100	0	0	0	11.23	1	3	0
10	81.4	28.6	18.6	10				8.0
20	62.9	57.1	37.1	20				14.8
30	44.3	85.7	55.7	30				20.6
40	25.7	114.3	74.3	40				25.8
50	7.1	142.9	92.9	50				30.3

^1^ CSR contents were controlled and shown in units of phr (parts per hundred resin).

## Data Availability

Data is contained within the article.
